# Rats undernourished in utero have altered Ca^2+^ signaling and reduced fertility in adulthood

**DOI:** 10.14814/phy2.12587

**Published:** 2015-10-27

**Authors:** Humberto Muzi-Filho, Alessandro M Souza, Camila G P Bezerra, Leonardo C Boldrini, Christina M Takiya, Felipe L Oliveira, Renata T Nesi, Samuel S Valença, Ananssa M S Silva, Gisele Zapata-Sudo, Roberto T Sudo, Marcelo Einicker-Lamas, Adalberto Vieyra, Lucienne S Lara, Valeria M N Cunha

**Affiliations:** 1 Institute of Biomedical Sciences, Federal University of Rio de JaneiroRio de Janeiro, Brazil; 2 Carlos Chagas Filho Institute of Biophysics, Federal University of Rio de JaneiroRio de Janeiro, Brazil; 3 National Institute of Science and Technology for Structural Biology and BioimagingRio de Janeiro, Brazil; 4 Directorate of Metrology Applied Life Sciences, National Institute of Metrology, Quality and TechnologyDuque de Caxias, Brazil

**Keywords:** Intrauterine malnutrition, oxidative damage markers, reproductive performance, vas deferens calcium handling

## Abstract

Epidemiological and animal studies have shown that placental undernutrition impairs reproduction in adult offspring, but the underlying molecular mechanisms within the male genital tract remain unknown. Due to its special physiological characteristics in transport and the modulation of the environment to which its luminal content is exposed, we hypothesized that the vas deferens would be a highly sensitive target. The goals were to investigate whether intrauterine malnutrition affects molecular mechanisms related to Ca^2+^- and oxidative stress-modulated processes and causes structural alterations in the adult rat vas deferens that could attenuate fecundity and fertility. Male adult rats malnourished in utero had increased vas deferens weight associated with thickening of the muscular coat, a decrease in the total and haploid germ cells, a marked increase in the immature cells, and a decline in the numbers of pregnant females and total offspring per male rat. The ex vivo response of vas deferens from malnourished rats demonstrated an accentuated decrease in the contractile response to phenylephrine. The vas deferens had a marked decrease in Ca^2+^ transport due to the uncoupling of Ca^2+^-stimulated ATP hydrolysis and ATP-driven Ca^2+^ flux, and the downregulation of both sarco-endoplasmic reticulum Ca^2+^-ATPase 2 and the coupling factor 12-kDa FK506-binding protein. An increase in protein carbonylation (a marker of oxidative damage) and an imbalance between protein kinases C and A were observed as a legacy of undernutrition in early life. These results provide the structural and molecular basis to explain at least in part how maternal undernutrition affects fecundity and fertility in adult male rats.

## Introduction

Intrauterine malnutrition is associated with an increased risk of developing cardiovascular, renal, and metabolic diseases in adulthood, as postulated in Barker’s programming hypothesis regarding the developmental origin of adult disease (Barker et al. 1993; Hoy et al. 1999; Vickers et al. 2000; Falkner 2002). Epidemiological and clinical studies support the model of the developmental origin of health and disease (Gluckman and Hanson 2004), in which individuals challenged by unfavorable environments acquire modified phenotypes to overcome the same situations in adulthood (the “predictive adaptive response”) (Hayward et al. 2013). If this is the case for in utero malnutrition (as an example of environmental defiance) and reproduction (as an example of a function to be maintained), compensatory changes in key organs must occur to preserve long-term survival and the preservation of the species. Thus, the driving hypothesis of this study is that in utero undernutrition programs alterations in the vas deferens that culminate in an abnormal phenotype in the adult associated with Ca^2+^- and oxidative stress-modulated processes.

Malnutrition jeopardizes the reproductive development and function of experimental animals and humans at different stages of life (Bergendahl and Veldhuis 1995; Chavatte-Palmer et al. 2008; Toledo et al. 2011; Hayward et al. 2013). The major effects are related to the impairment of the hypothalamic–pituitary–gonadal axis (Genovese et al. 2010). This impairment, which is followed by secondary sex organ atrophy, such as in the testicular structure accompanied by a decrease in Sertoli cells, ultimately leads to lower daily sperm production in adults (Genovese et al. 2010).

Although there is a general understanding of the links between nutritional status and sex hormone regulation, little is known about the mechanisms that lead to the dysfunction of male reproductive organs, and much less is known regarding possible adaptive responses. In this context, the vas deferens deserves special attention because it tightly regulates the luminal environment to which sperm cells are exposed (Pierucci-Alves et al. 2009, 2010) during their storage (Peirce et al. 2003) and passage from the epididymis to the urethra (Westfall and Westfall 2001; Koslov and Andersson 2013). For these reasons, alterations in this organ – which is less investigated than others in the genital tract – are critical for fertility impairment (Sato et al. 2005; Chen et al. 2012). Thus, in the context of the programming hypothesis, it is plausible that prenatal undernutrition induces molecular alterations in the vas deferens that can compromise reproductive success in adulthood, even with normal nutrition postpartum and later. The denervation and disruption of the complex formed by the ryanodine-sensitive Ca^2+^ release channel and the 12-kDa FK506-binding protein (FKBP12) in the vas deferens result in changes in vas deferens contractility (Quintas et al. 2005; Scaramello et al. 2009). Therefore, it is likely that alterations in Ca^2+^ signaling by the vas deferens are involved in impaired reproductive function in male rats (Thomas 1983; Scaramello et al. 2009).

We have demonstrated that multifactorial chronic undernutrition cripples the reproductive capacity of young adult rats (Muzi-Filho et al. 2013), which led us to investigate whether maternal undernutrition during a narrow window of gestation affects the reproductive axis of adult male offspring or induces potentially compensatory benefits on fertility and fecundity. We also posited that molecular and structural alterations in this organ would impair reproduction in adult life, even when normal nutrition is restored after birth. We induced malnutrition with a handmade diet to mimic a diet common among impoverished populations in northeast Brazil, which we refer to as the regional basic diet (RBD); this diet is also representative of other basic food patterns worldwide (Teodósio et al. 1990). The RBD induces several metabolic and physiological changes (growth, water distribution among body fluid compartments, and renal and cardiovascular functions) in adult young animals that in many cases seem to be due to the maternal nutritional status (Paixão et al. 2005; Luzardo et al. 2011; Vieira-Filho et al. 2014).

The goals of our study were achieved by showing altered Ca^2+^ transport and Ca^2+^-dependent ATP hydrolysis, decreased abundance of sarco-endoplasmic reticulum Ca^2+^-ATPase (SERCA), downregulation of FKBP12, imbalanced 4 protein kinase C (PKC)/AMP-dependent protein kinase (PKA) activities, and increased protein carbonylation without lipid peroxidation. At the cellular, structural, and functional levels, maternal undernutrition leads to thickening of the vas deferens muscle coat, the persistence of immature cells in the testes and throughout the reproductive tract, a marked decrease in the contractile response of the vas deferens to the *α*1 receptor agonist phenylephrine, and a decline in fertility and fecundity during adulthood.

## Methods

### Ethical considerations

Procedures were conducted according to “The Guide for Care and Use of Laboratory Animals” (DHHS Publication No. [NIH] 85-23) and were approved by the Ethics Committee on Animal Use at the Federal University of Rio de Janeiro (protocol CEUA DFBCICB 007).

### Diets and animals

The deficient diet (RBD) was formulated and prepared using the dietary recall method; it is representative of a diet consumed in impoverished areas of cities from developed countries and in >30 developing countries in Africa, the Middle East, and Central and South America (McLaren and Pellett 1970; Murillo et al. 1974; Pak and Araya 1977; Ramos-Aliaga 1978), where basic food patterns are similar to those encountered in Brazilian regions where RBD – or a similar diet – is widely consumed. The composition of the RBD was established by Teodósio et al. (1990) in the late 1980s after performing an epidemiological survey in different northeast Brazilian regions, which included income, food availability, and nutritional habits from one of the three physiographic zones of Pernambuco State in the South “Mata” area. It is worth mentioning the original reason that led Teodósio et al. to use this diet, which explains why its composition is prepared in the laboratory and is not an industrial formulation. The RBD was formulated according to the data from 918 families (5939 persons), and the original procedures of preparation were developed by the Laboratório de Nutrição em Saúde Pública of their Department using the dietary recall method. Taking into account their highest consumption and frequency, lowest cost, availability in the market, and use in the main daily meal, four foods were selected: brown beans (*Phaseolus vulgaris*), manioc flour (*Manioc esculenta*), dried and salted meat, and sweet potato (*Ipomoea batatas*). These four foods were used in the RBD in the proportions described in the surveys. Thus, the deficient diet was formulated with specific and natural ingredients used worldwide, which more closely reflects the actual diet than commercial preparations in which the percentages of one or more components are lower. For this reason, the ingredients were different than those used in normal chow. When populations were compared, one group consumed a normal diet according to the cultural alimentary habits, and the other consumed a deficient diet as used in the present study. Although certain factors, such as phytoestrogen activity, could be different in the on-site prepared diet, no industrial chow can exactly duplicate the diet consumed by a population that predominantly has access to only a few ingredients, such as beans, manioc flour, jerked meat, and sweet potatoes.

Following the original formulation, the RBD we used had the following relative composition (w/w): 18% beans (*P. vulgaris* or brown beans), 65% manioc flour, 4% jerked meat, 0.35% fat separated from the original crude meat, and 13% sweet potatoes. These ingredients were cooked separately, dehydrated at 60°C, and ground. They were mixed, and water was added to form a wet mass, which was cut into small pieces with a shape similar to that found in the standard chow diet before being finally dehydrated for 1 day at 60°C. This preparation of RBD provided the nutrients detailed in Table1. Notably, the diet was deficient in proteins; >90% was provided by beans, and <10% was provided by meat. The RBD is poor in minerals in quantitative contrast to the control (CTRL) diet (Table1; Teodósio et al. 1990; Vieira-Filho et al. 2009). A qualitative deficiency in the source of energy was also noted. The total energy supply in RBD was similar to that found in normal chow (∼310 kcal/100 g dry weight vs. ∼280 kcal/100 g dry weight); however, only ∼1% was lipidic in origin, contrasting with ∼10% in the control diet. Most of the calories in RBD was provided by its higher carbohydrate content (sweet potatoes and manioc flour). Finally, vitamins (such as ascorbic acid, retinol, biotin, thiamin, riboflavin, niacin, para-aminobenzoic acid, pyridoxine, inositol, cyanocobalamine, and choline) were present at very low levels, as calculated by Teodósio et al. (1990) based on the vitamin content of the ingredients (Sebrel and Harris 1954). The control diet given to normo-nourished dams during gestation was purchased from Purina Agribands (Paulínia, Brazil). This chow was supplemented during manufacturing with vitamins as established in the AIN-93G requirements (Reeves et al. 1993). The respective chows and water were offered daily ad libitum to both groups of mothers.

**Table 1:** Composition of diets

	CTRL^1^	RBD^2^
Protein % (w/w)	23	8
Carbohydrate % (w/w)	41	78
Lipids % (w/w)	2.5	1.7
Na % (w/w)	0.3^3^	0.2^3^
Fe % (w/w)	0.018	0.007
Ca % (w/w)	1.8	0.04
K % (w/w)	0.9	0.3
Energy supply kcal/100 g dry weight	278	356
Vitamin supplement	Yes	No

CTRL, control; RBD, regional basic diet.

1 As indicated by the manufacturer (Purina Agriband, Paulínia, Brazil).

2 According to the Laboratory of Experimentation and Analysis of Food (LEEAL), Nutrition Department, Federal University of Pernambuco.

3 According to Silva et al. (2014a,b).

The fresh ingredients were purchased weekly in a grocery exhibiting an official health certificate. They were immediately used to prepare RBD following rigid food fabrication safety practices established in the Laboratory of Analysis and Food Processing (Nutritional Biochemistry Research Group, dgp.cnpq.br/dgp/espelhogrupo/4898598097876684), Institute of Nutrition Josué de Castro, Federal University of Rio de Janeiro. RBD was always prepared as required within 7 days of use and stored in sealed plastic containers at 4°C that were carefully cleaned before and after storing the food.

Animals were housed at 25 ± 1°C on a 12:12-h light–dark cycle. Fifteen female Wistar rats (aged 12* *weeks) were randomly mated on the same day through a HAREM method with five healthy Wistar males of the same age (∼350 g) that came from different litters in the vivarium. Eight dams selected at random were subjected to undernutrition by feeding RBD throughout gestation (the intrauterine malnourished [IM] group) immediately after pregnancy was confirmed by the daily analysis of the vaginal plug, whereas six dams continued eating the control diet (the CTRL group). The first day of gestation was when spermatozoids were found in the vaginal plugs. After the pups’ birth, both groups of mothers were fed standard rat chow throughout lactation. The eight and six litters, respectively, were maintained with eight pups (including female pups if necessary) from day 2 after birth to guarantee a similar number of pups for breast feeding. After weaning, three to four male rats from the same litter were allocated into the same cage (total of nine cages for each group, see Table3). All litters were represented in both experimental groups because one male from each cage was randomly selected to compose the “*n*” indicated in Table3. When the offspring reached 25 days (the end of weaning), the normo-nourished control (CTRL) and IM rats were fed with standard diet until early adult life (13 weeks). Food and water were offered ad libitum. After analyzing the reproductive profile in vivo, the rats were killed by decapitation, and their reproductive organs were removed to perform the following tests: (1) determine the cell distribution in the testis, epididymis, and vas deferens; (2) provide histomorphometry of the vas deferens; and (3) calculate the *index* of the organs (testis, epididymis, or vas deferens weight/body weight ratio). The impacts of the multifactorial diet on the mothers (body weight on day 20 of gestation, total weight gain, total dietary intake during gestation, total energy intake, number of fetuses, and placental weight) and the pups (fetal weight on day 20 of gestation, body weight at birth and weaning) are presented in Table2.

**Table 2:** General characteristics of mothers and pups

Measurement	CTRL	IM
Maternal data^1^		
Body weight on day 20 of gestation (g)	347 ± 9 (6)	242 ± 9* (7)
Total weight gain (g)	112 ± 6 (6)	4.9 ± 10.8* (7)
Total dietary intake during gestation (g)	394 ± 15 (6)	302 ± 7* (7)
Total energy intake (kcal)	1096 ± 41 (6)	1136 ± 25 NS (7)
Number of fetuses	12.8 ± 0.8 (6)	8.1 ± 1.1* (7)
Placental weight (g)	0.40 ± 0.01 (6)	0.34 ± 0.02* (7)
General data of fetuses and newborn pups		
Fetal weight on day 20 of gestation (g)^2^	2.27 ± 0.04 (7)	2.02 ± 0.05* (8)
Body weight at birth (g)^3^	6.4 ± 0.1 (13)	5.4 ± 0.2* (16)
Body weight at weaning (g)^3^	66.2 ± 1.6 (5)	56.9 ± 1.2* (4)

The data are the mean ± SEM. Number of animals in parentheses. CTRL, control; IM, intrauterine malnourished; NS, not significant.

1 From Vieira-Filho et al. (2009).

2 Pups from different dams.

3 Each rat from different litters.

* *P *<* *0.05 versus CTRL assessed by unpaired Student’s *t*-test.

In the series of functional experiments aiming to investigate the impact of maternal undernutrition on vas deferens contractile activity, an additional two CTRL and two IM mothers were used. Three or four CTRL and IM male offspring from the different litters (*n *=* *2 for each experimental group) were selected at random and solely employed to evaluate the response to phenylephrine of isolated strips of vas deferens at 13 weeks of age.

### Preparation of vas deferens homogenates

Vas deferens homogenates were prepared as described previously (Scaramello et al. 2009; Muzi-Filho et al. 2013). After weighing and decapitation, the vasa deferentia were identified after the longitudinal dissection of the lower abdominal region and the careful removal of the neighboring fat. The whole duct between the prostate and the epididymis was collected and placed in ice-cold Tyrode’s solution (137 mmol/L NaCl, 2.7 mmol/L KCl, 11.9 mmol/L NaHCO_3_, 0.36 NaHPO_4_, 5.5 mmol/L glucose, 1.8 mmol/L CaCl_2_, and 0.4 mmol/L MgCl_2_) equilibrated with a gas mixture of 95% O_2_ and 5% CO_2_ at pH 7.4 and room temperature. After carefully removing the peripheral adipose tissue, the vas deferens was perfused three times with this solution using an insulin syringe. Two or three pairs of organs removed from rats from different litters were suspended in solution (250 mmol/L sucrose, 5.5 mmol/L Tris-HCl at pH 7.4, 2 mmol/L dithiothreitol, 0.2 mmol/L phenylmethylsulfonyl fluoride, 1 mmol/L ethylenediamine-tetraacetic acid (EDTA), 2 *μ*g/mL antipain, 5 *μ*g/mL aprotinin) for homogenization in an Ultraturrax disperser (IKA Works, Campinas, Brazil) at 20,500 rpm for 60 sec followed by two additional spins of 30 sec at an interval of 20 sec. The suspension was centrifuged at 108,000*g* for 60 min at 4°C, and the sediment was resuspended in 250 mmol/L sucrose for storage in liquid N_2_. The protein content was determined using the Folin method (Lowry et al. 1951).

The biochemical experiments performed with these homogenates required stocks in the range of 10 mg/mL of protein and volumes of 2–3 mL for single assays in triplicate or quadruplicate. Thus, to achieve these quantities, two or three pairs of vas deferens from rats originating from different litters were pooled and used as the starting material for homogenization, thus attenuating variations due to litter effects. The use of four preparations obtained in this manner ensured that all litters were represented.

### Investigation of vas deferens Ca^2+^ handling

Ca^2+^ uptake experiments were performed at 37°C in medium containing ^45^CaCl_2_ (GE Healthcare Bio-Sciences, Pittsburgh, PA) (specific activity ∼1.5 × 10^9^ Bq/mmol), 10 *μ*mol/L free Ca^2+^ calculated as per Sorenson et al. (1986), 0.3 mmol/L ethylene glycol-bis(β-aminoethyl ether)-N,N,N′,N′-tetraacetic acid (EGTA), 60 mmol/L KCl, 50 mmol/L 3-(*N*-morpholino) propanesulfonic acid-Tris (MOPS-Tris) (pH 7.4), 10 mmol/L NaN_3_, 4 mmol/L MgCl_2_, and 5 mmol/L ATP (disodium salt). The reaction was started by adding 80 *μ*g protein from the vas deferens homogenates to the medium and stopped 2 h later by rapid filtration. Nitrocellulose filters were washed twice with 10 mL cold medium (2 mmol/L LaNO_3_, 100 mmol/L KCl, and 20 mmol/L MOPS-Tris [pH 7.0]), dried, and counted. Specific Ca^2+^ uptake was calculated by subtracting the uptake measured in the absence of 5 mmol/L ATP (blanks) from the total uptake. These blanks taken as negative controls were obtained by incubating the membranes with ^45^Ca without ATP. In this combination, the radioactivity recovered in filters was <10%. Positive controls included ^45^Ca uptake by sarcoplasmic reticulum vesicles obtained from rabbit muscle and vesicles derived from renal basolateral membranes in a medium similar to that described by Sola-Penna et al. (1994). The experiments were repeated four times (with triplicate samples) using different vas deferens membrane preparations.

Ca^2+^-ATPase activity (from plasma membrane, PMCA, and SERCA) was assayed as described previously (Scaramello et al. 2009; Muzi-Filho et al. 2013). The reaction medium was the same as that used for Ca^2+^ uptake experiments, except that unlabeled CaCl_2_ replaced ^45^CaCl_2_, there were three different free Ca^2+^ concentrations (1 nmol/L, 100 nmol/L, and 10 *μ*mol/L), the reaction times were 5 and 120 min, and [*γ*-^32^P]ATP (specific activity ∼1.5 × 10^10^ Bq/mmol; prepared as per Maia et al. 1983) was used instead of unlabeled ATP with or without 3 *μ*mol/L thapsigargin (GE Healthcare Bio-Sciences) as the specific inactivator of SERCA (Sagara and Inesi 1991). Reactions were stopped by adding 1 mL cold 26% (w/v) charcoal in 0.1 N HCl, and the tubes were centrifuged at 1500 *g* for 15 min at 4°C so that 250 *μ*L of the supernatant could be counted. Ca^2+^-ATPase activity was determined by subtracting the ^32^P_i_ released in the absence of CaCl_2_ (0.3 mmol/L EGTA). SERCA activity was measured by the difference between total and thapsigargin-resistant Ca^2+^-ATPase activity (PMCA). The negative controls were tubes run in parallel, in which a small amount of ^32^P_i_ was released during the incubation period from [*γ*-^32^P]ATP in the absence of membranes. The positive controls were Ca^2+^-ATPase activities from the same membranes used in Ca^2+^ uptake experiments. Ca^2+^-ATPase experiments were repeated five times (with triplicate samples) using different membrane preparations.

Sodium dodecyl sulfate polyacrylamide gel electrophoresis (SDS-PAGE) was performed by the method of Laemmli (1970). The homogenates (80 *μ*g protein) were separated (60 mA per gel, 60 min) and transferred to nitrocellulose membranes (350 mA for 90 min). The blotted membranes were blocked with 5% non-fat milk containing 0.1% Tween 20 (60 min) and then incubated overnight with specific 1:1000 diluted antibodies (Santa Cruz Biotechnology, Santa Cruz, CA, for PMCA and FKBP12; Sigma-Aldrich, Saint Louis, MO, for SERCA2). The blots were developed after 60 min incubation with anti-rabbit (PMCA and FKBP12; 1:10,000) or anti-mouse (SERCA2) IgG horseradish peroxidase-conjugated antibodies, and a Hyperfilm-ECL kit (GE Healthcare Bio-Sciences) was used to detect the bands. The catalog numbers of the primary antibodies are given in the corresponding figure legends. Immunoanalyses were performed using three to four different vas deferens homogenates; samples from CTRL and IM homogenates were analyzed in parallel in the same gel. Densitometric values were corrected for protein loading using Ponceau red staining because the *β*-actin content was lower in the vas deferens from the IM group. After electrophoresis and transfer, the nitrocellulose membrane was stained with Ponceau red (Sigma), and immunodetection was performed with a specific antibody. After the identification of the protein under study, the images were superimposed with the mass-matched scanned Ponceau red images, and the intensity ratios were calculated. The results are thus given as the ratio of the immunodetection intensity signal to the protein loading, which was expressed as percentage of the corresponding CTRL ratio. The mean intensities of the normalized immunosignals from CTRL rats were considered to be 100%, and all data from CTRL and IM rats were expressed as percentages of this reference value, thus allowing the calculation of the SEM in CTRL conditions (Sahajpal and Ashton 2005; Silva et al. 2014b).

### Oxidative stress markers in the vas deferens

Lipid peroxidation was assessed in freshly removed whole vas deferens by measuring thiobarbituric acid reactive species (Buege and Aust 1978) with slight method modifications (Vieira-Filho et al. 2011, 2014; Muzi-Filho et al. 2013). The results have been expressed in *μ*mol malondialdehyde/mg of vas deferens. Protein carbonylation was assayed in homogenates according to Menegali et al. (2009) with slight modifications (Muzi-Filho et al. 2013). The total free sulfhydryl group content (-SH) was also measured in homogenates (Ellman 1959; Herken et al. 2009). The results are expressed as *μ*mol total free sulfhydryl groups/mg vas deferens protein.

### Kinase-mediated signaling in vas deferens

The abundance of the cyclic PKA *α*-catalytic subunit and PKC isoforms in the vas deferens was measured by western blotting (as above) using 1:500-diluted primary (Santa Cruz Biotechnology) and 1:5000-diluted secondary anti-rabbit antibodies. Kinase-mediated activity was determined by the incorporation of the *γ*-phosphoryl group of [*γ*-^32^P]ATP into histone in the absence or presence of 10 nmol/L PKAi_5-24_ (PKA specific inhibitor) or 10 nmol/L calphostin C (a specific inhibitor of classical and novel PKC isoforms), as described previously (Cabral et al. 2007; Vieira-Filho et al. 2011; Muzi-Filho et al. 2013). Negative controls for kinase-mediated activities were samples in which histones were incubated with [*γ*-^32^P]ATP in the absence of vas deferens membranes, resulting in a minimally detectable signal of radioactivity in the nitrocellulose filters. Positive controls included PKA and PKC activities measured with the use of purified renal basolateral membranes, in which the kinases were detected (Cabral et al. 2010; De Souza et al. 2010; Axelband et al. 2012). Immunodetection was performed using different membrane preparations (three for PKA and four for each PKC). The activities were assayed in quadruplicate using four different membrane preparations.

### Histological analysis

Anesthetized rats (ketamine/xylazine 5/50 mg per kg body weight, respectively, from Cristália, Itapira, Brazil and Bayer, Belford Roxo, Brazil) were perfused via the left cardiac ventricle with 4% (w/v) phosphate-buffered paraformaldehyde (pH 7.4) containing heparin (10 U/mL). The vasa deferentia were removed, dissected, and transversely sectioned in their epididymal (initial first quarter) and prostatic (final quarter) segments. These were fixed in 10% formaldehyde for 24 h, dehydrated in ethanol (70%, 95%, and 100%), clarified in xylene, and embedded in paraffin. For histomorphometric studies, four sections of 5-*μ*m thickness were obtained from each portion of six rats from different litters and stained with Masson’s trichrome. Images of each section were taken with a digital camera (Media Cybernetics, Rockville, MD) coupled to a light microscope (Nikon, Tokyo, Japan). Thirty high-quality images (2048 × 1536 pixels buffer) from each section were examined with Pro Plus 4.5.1 software (Media Cybernetics) (Beiral et al. 2014). The thickness (*μ*m^2^) of the muscular coat area – calculated as the difference between the total area of the section and the luminal area – was always blindly evaluated by the same person (L. C. B.). The total area was chosen instead of the distances from the tunica adventitia to the epithelial layer for two reasons (1) the distances are not exactly equal in the same section and (2) the measurement of area incorporates all linear distances.

### Total and haploid cell count

Sperm cells were counted according to a previous method (Joyce et al. 1993) with slight modifications (Muzi-Filho et al. 2013), and the DNA content was measured (Vindeløv et al. 1983). Testis, whole epididymis, or the vas deferens (∼300 mg) were suspended in 1 mL of a solution containing 150 mmol/L NaCl supplied with 0.05% (v/v) Triton X-100 and smoothly homogenized by hand for 1 min using a glass Potter homogenizer and a Teflon pestle. Control microscopic analysis in a Neubauer chamber (hemocytometer) was performed in all experiments. Using Trypan blue, we observed that cell viability and sperm cell motility were preserved during this mild detergent treatment and smooth homogenization. These cell suspensions were then incubated in 1 mL of the Vindelov solution containing 0.1% (v/v) Na-citrate buffer, 0.1% (v/v) Triton X-100, 0.5 g/mL RNAse, and 50 *μ*g/mL propidium iodide (Sigma-Aldrich). After a 15-min incubation, the 1-mL suspension was counted by flow cytometry (FACSCalibur Flow Cytometer, Becton Dickinson, Franklin Lakes, NJ) using Cell-Quest software, and the DNA content was measured by WinMDI 2.9 (The Scripps Research Institute, La Jolla, CA). Considering the DNA content, cells were classified as total, haploid (G0-G1 phase, *n*), immature (S phase [>*n* but <2*n*]), and diploid in G2/M phase (2*n*). This notation implies the following: haploid cells include secondary spermatocytes, spermatids, and spermatozoa; immature corresponds to cells in division; and diploid cells correspond to spermatogonia and primary spermatocytes (Kotaja 2014). The data have been expressed as counts × sec^−1^ per 100 mg wet weight of whole testis, epididymis, or vas deferens; these counts were obtained from the number of events provided by the flow cytometer during an acquisition time of 10 sec, which is sufficient for a flow cytometer to acquire 100 *μ*L of the cell suspension. Subsequently, this scale was adjusted to cells/mL. Cell dissociation did not affect fluorescence under our experimental conditions.

### Contractile activity of the epididymal portion of the vas deferens

The contractile activity of strips from the isolated epididymal portions of CTRL and IM vas deferens was assayed as described by Scaramello et al. (2009). Briefly, the organs were placed in vertical baths filled with a modified Tyrode solution (138 mmol/L NaCl, 5.7 mmol/L KCl, 15 mmol/L NaHCO_3_, 0.36 mmol/L NaH_2_PO_4_, 5.5 mmol/L glucose, 1.77 mmol/L CaCl_2_) at 30°C bubbled with a carbogenic mixture (95% O_2_, 5% CO_2_). After a stabilization period of 45 min under a tension of 1 g, contractile responses were elicited by repeated additions of concentrated KCl at 45-min intervals to a final concentration of 80 mmol/L until the contractions were reproducible. Then, increasing phenylephrine concentrations (0.3–100 *μ*mol/L) were added with interval washout periods of 30 min to generate the dose–response curve, which was used to calculate the maximal contractile effect (E_max_) and the effective phenylephrine concentration that corresponds to 50% of the maximal response (EC_50_). The signals generated by the force transducer (MLT0201; ADInstruments, Dunedin, New Zealand) were amplified using Quad Bridge Amp (ADInstruments) and digitized (PowerLab 8/35 PL 3508 model; ADInstruments) for further analysis. The final analysis of the data was performed with LabChart 7.3.1 (module Dose-Response; ADInstruments).

The parameters of the pharmacological response were calculated from the following equation (Nojimoto et al. 2009) adjusted to the experimental points:

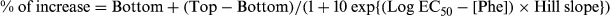
1where Bottom is the asymptotic lower value of the curve, Top is the asymptotic upper value of the curve, EC_50_ is the phenylephrine concentration that elicits half-maximal stimulation, [Phe] is the micromolar concentration of phenylephrine, and Hill slope quantifies the steepness of the curve.

### Reproductive profile analysis

The reproductive performance of male rats (fertility and fecundity) was measured as in a previous report (Muzi-Filho et al. 2013). Controls for female contribution to impaired reproductive capacity were also performed. Virgin female normo-nourished rats aged 13 weeks were mated by the HAREM method (one CTRL or IM male with three females) and housed for 10 days at 25°C under a 12:12-h light–dark cycle, receiving standard chow and drinking water ad libitum, to cover 2–3 periods of estrus. The number of living pups per female was counted and averaged 10 pups per mother, regardless of whether the females were mated with a CTRL or an IM male. We were not aware, before mating, whether all females were fertile or not. After a period of 21–34 days from the first day of mating (depending of the moment of conception detected by the daily assessment of the vaginal plug), fertility (number of pregnant females per one male) and fecundity (number of pups per one male) were evaluated.

### Statistics

Except where otherwise indicated, the data are presented as the mean ± SEM. Differences between parameters in the CTRL and IM groups were analyzed using unpaired Student’s *t*-tests. Multiple comparisons were made by one-way analysis of variance (ANOVA) followed by the Bonferroni post-test for selected pairs. Adult body weight and organ weights (testis, epididymis, and vas deferens) were further analyzed by analysis of covariance (ANCOVA) with body weight at birth as a covariate. *P *<* *0.05 was considered significant in all analyses. Statistical tests and graphs used GraphPad Prism 6.0 software (GraphPad Inc., La Jolla, CA). The “*n*” values are shown in the corresponding figure legends and tables.

## Results

### General data

Table3 shows that male rats malnourished in utero (IM group) had marked alterations in their reproductive tracts by adulthood. First, it is remarkable that they weighed less despite being normally fed at weaning and thereafter, and the difference in body weight correlated significantly with the lower body weight at birth (an imprinting of the maternal undernutrition), as revealed by ANCOVA. Second, there was no difference in the testis weight, a parameter that was also not influenced by the lower birth body weight; the increased testis index thus reflects the decreased corporal mass. Third, their reduced epididymal mass corresponded with their decrease in total body mass. Fourth, the vas deferens of the IM group increased in absolute mass by 11%, a difference that became exaggerated to 20% after normalization to the corresponding reduced body mass. The lower birth weight impacted the mass of both the epididymis and the vas deferens despite its opposite effects on these parts of the reproductive tract.

**Table 3:** Body weight, organ (testis, epididymis, and vas deferens) wet weight, and organ/body weight ratio

Measurement	CTRL (*n *=* *9)	IM (*n *=* *9)	*P* value^1^	ANCOVA	
				R^2^	*P* value^2^
Body weight (g)	405 ± 2	376 ± 2	<0.0001	0.8312	<0.0001
Testis weight (mg)	1599 ± 14	1627 ± 15	NS	0.1080	NS
Testis index (mg/g)	3.94 ± 0.04	4.32 ± 0.05	<0.0001	–	–
Epididymis weight (mg)	390 ± 7	347 ± 4	<0.0001	0.6521	<0.0001
Epididymis index (mg/g)	0.96 ± 0.02	0.92 ± 0.01	NS	–	–
Pair of vas deferens weight (mg)	110 ± 2	122 ± 3	0.002	0.4069	0.0044
Vas deferens index (mg/g)	0.27 ± 0.004	0.32 ± 0.009	<0.0001	–	–

The data are the mean ± SEM. ANCOVA, analysis of covariance; CTRL, control; IM, intrauterine malnourished; NS, not significant.

1 Statistical significance (IM vs. CTRL) was assessed by unpaired Student’s *t*-test.

2 ANCOVA was applied using birth weight (Table2) as a covariate.

### Intrauterine malnutrition imprints Ca^2+^ handling abnormalities in the vas deferens

**Figure 1:**
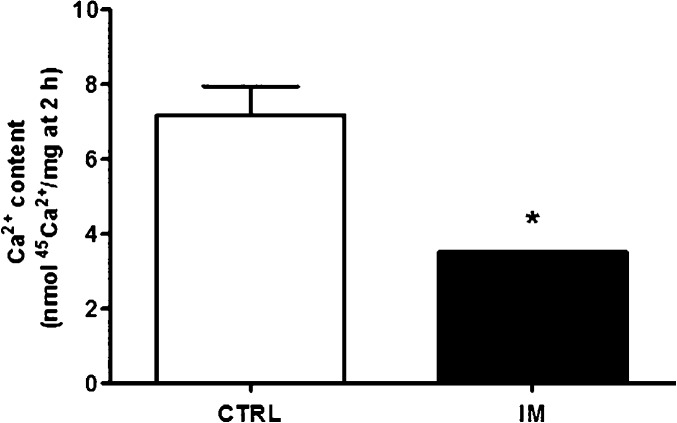
Intrauterine malnutrition reduces Ca^2+^ accumulation by vesicles derived from vas deferens membranes. Steady-state ATP-dependent Ca^2+^ accumulation in the lumen of vesicles derived from membranes of whole vas deferens homogenates was measured in control (CTRL) and intrauterine malnourished rats (IM) after 120 min. The results are the mean ± SEM (*n *=* *4 homogenates obtained with 2–3 pairs of vas deferens of rats from different litters); *P =* *0.0018 compared with the CTRL group (assessed by unpaired Student’s *t*-test).

**Figure 2:**
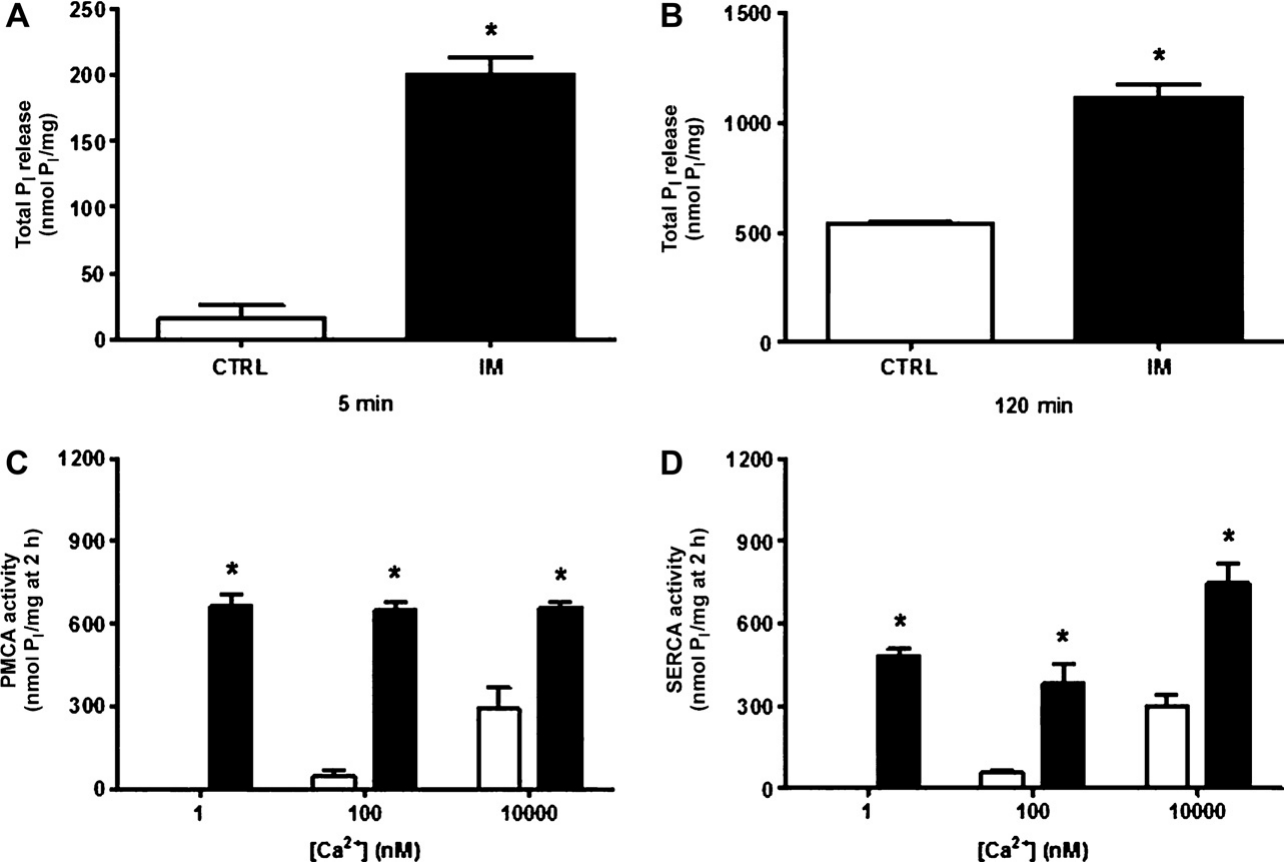
Fetal malnutrition increases the response to Ca^2+^ for PMCA and SERCA activities from the vas deferens. (A) Initial rate of total P_i_ release: Ca^2+^-ATPase activity was measured at 5 min in homogenates from CTRL (empty bars) and IM rats (black bars). The results are the mean ± SEM (*n *=* *3–5 homogenates obtained as described in the legend to Fig.1); *P <* *0.0001 with respect to CTRL. (B) Total P_i_ release at the time of Ca^2+^ uptake measurements (120 min). Groups and symbols as in (A). The results are the mean ± SEM (*n *=* *3–5 homogenates obtained as described in the legend to Fig.1); *P =* *0.0002 with respect to CTRL. Differences in (A) and (B) were assessed by unpaired Student’s *t*-test. (C and D) Activity of PMCA and SERCA were measured at 120 min in the presence of the Ca^2+^ concentrations shown. CTRL: empty bars; IM: black bars. The results are the means ± SEM (*n *=* *3–5 homogenates as above). In (C and D): *P <* *0.0001 in all cases compared to the corresponding CTRL at 1, 100, and 10,000 nmol/L Ca^2+^, respectively. Differences were assessed by one-way ANOVA followed by the Bonferroni post-test for selected pairs. PMCA, plasma membrane Ca^2+^-ATPase; SERCA, sarco-endoplasmic reticulum Ca^2+^-ATPase; CTRL, control; IM, intrauterine malnourished; ANOVA, analysis of variance.

In utero malnutrition reduced the stationary Ca^2+^ accumulated in vesicles derived from whole homogenates of the vas deferens by ∼50% (Fig.1). This occurred in spite of the marked increase in both PMCA and SERCA activity, which were distinguished from total Ca^2+^-ATPase with thapsigargin to assess their respective contributions (Fig.2). Enhanced ATP hydrolytic capacity was observed either at 5 min (Fig.2A), when unidirectional Ca^2+^ fluxes predominated, or at 2 h (Fig.2B), when Ca^2+^ influx and Ca^2+^ efflux were in balance. Figure2C and D show that the undernutrition-induced increase in Ca^2+^-ATPase relied on an abnormal response to Ca^2+^; 1 nmol/L Ca^2+^, a concentration at which catalysis by the two enzymes was barely detectable in the CTRL group, gave maximal activation in the IM group.

**Figure 3:**
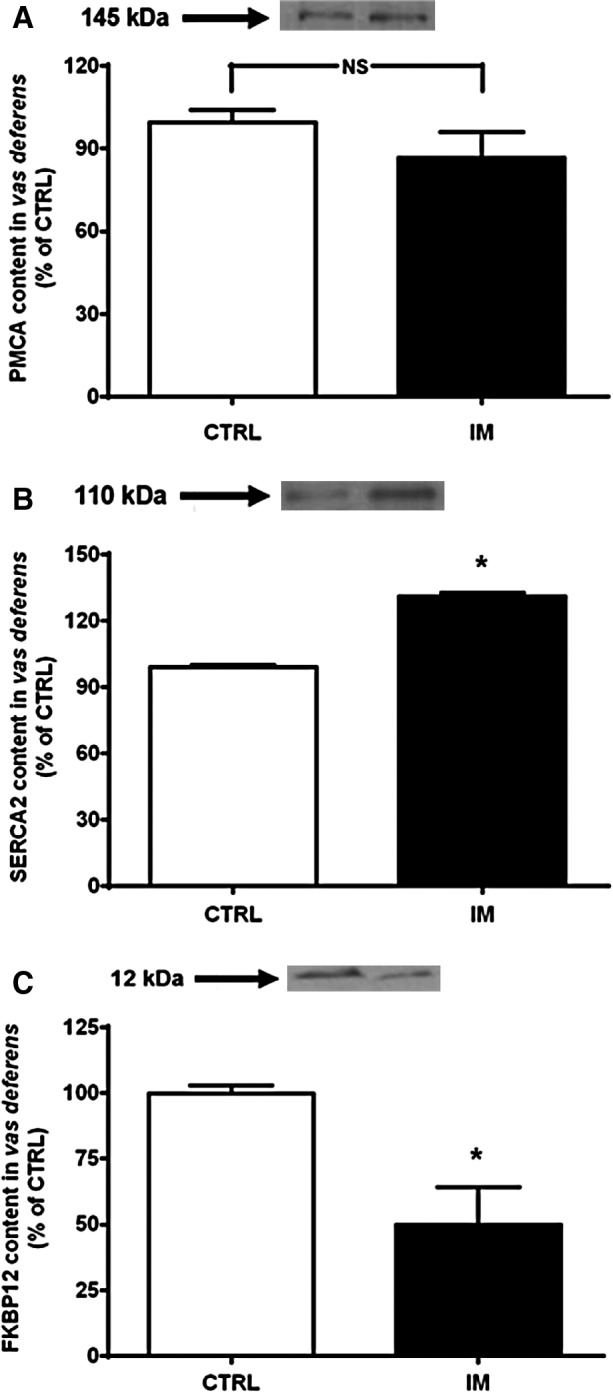
Undernutrition-induced modifications in the abundance of Ca^2+^-handling proteins (PMCA, SERCA2, and FKBP12) in the vas deferens. (A) PMCA. Upper panel: representative immunoblotting. Lower panel: graphic representation of four immunoanalyses performed using different preparations (homogenates obtained with 2–3 pairs of vas deferens of rats from different litters) and a primary antibody from Santa Cruz Biotechnology (catalog number sc-28765). Samples from CTRL and IM were analyzed in parallel in the same gel (10% acrylamide). (B) SERCA2. Upper panel: representative immunoblotting. Lower panel: graphic representation of four immunoanalyses (homogenates obtained with 2–3 pairs of vas deferens from rats from different litters) and a primary antibody from Sigma-Aldrich (catalog number s1314). *P =* *0.0005 versus the corresponding CTRL, as assessed by unpaired Student’s *t*-test. (C) FKBP12. Upper panel: representative image of three immunoblottings, which were performed using different preparations as above and a primary antibody from Santa Cruz Biotechnology (catalog number sc-28814). Lower panel: graphic representation of the three immunoanalyses. The data are expressed as the percentage of CTRL (mean ± SEM). *P =* *0.009 versus the corresponding CTRL (unpaired Student’s *t*-test). PMCA, plasma membrane Ca^2+^-ATPase; SERCA, sarco-endoplasmic reticulum Ca^2+^-ATPase; FKBP12, 12-kDa FK506-binding protein; CTRL, control; IM, intrauterine malnourished; NS, not significant.

The following experiments determined whether alterations in activity were related to PMCA and SERCA abundance. Although intrauterine malnutrition did not alter PMCA content (Fig.3A), SERCA2 was significantly upregulated by ∼35% (Fig.3B). Another important effect was a strong decrease in FKBP12 (Fig.3C), an immunophilin responsible for maintaining the intracellular Ca^2+^ channel in its closed conformation (Lanner et al. 2010; Okatan et al. 2013). This result is in agreement with the disrupted ability to retain Ca^2+^ inside vesicles derived from the membranes (Fig.1).

### In utero malnutrition causes protein oxidative damage in the vas deferens

**Figure 4:**
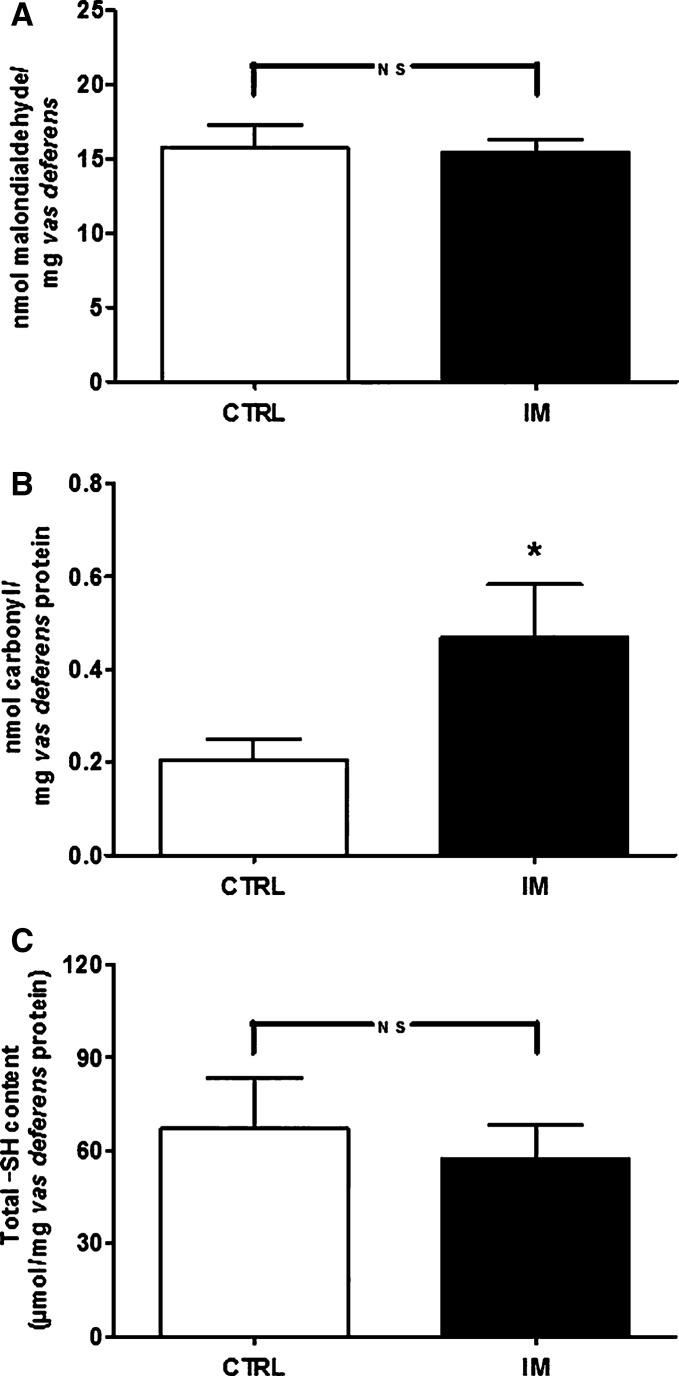
In utero malnutrition increases carbonylation but not lipid peroxidation or free sulfhydryl (-SH) content. (A) Levels of lipid peroxidation (*n *=* *3 pairs of vas deferens; all rats coming from different litters). (B) Protein carbonylation (*n *=* *6 homogenates obtained with 2–3 pairs of vas deferens of rats from different litters). (C) Total free -SH group content (*n *=* *3 homogenates as above). The results are expressed as the mean ± SEM. In B, *P =* *0.039 versus CTRL (unpaired Student’s *t*-test). CTRL, control; NS, not significant.

Intrauterine malnutrition induces increased tissue oxidative stress during early life (Magalhães et al. 2006), which gradually returns to normal with time (Vieira-Filho et al. 2011). In contrast to what is seen in chronically malnourished rats (Muzi-Filho et al. 2013), lipid peroxidation levels were unchanged in IM males (Fig.4A). Figure4B illustrates the imprint of oxidative damage on the vas deferens caused by malnutrition; there was a 2.5-fold increase in protein carbonylation. Thiol groups remained unmodified in this damage (Fig.4C).

### In utero malnutrition alters the content and activity of protein kinases A and C in the vas deferens

**Figure 5:**
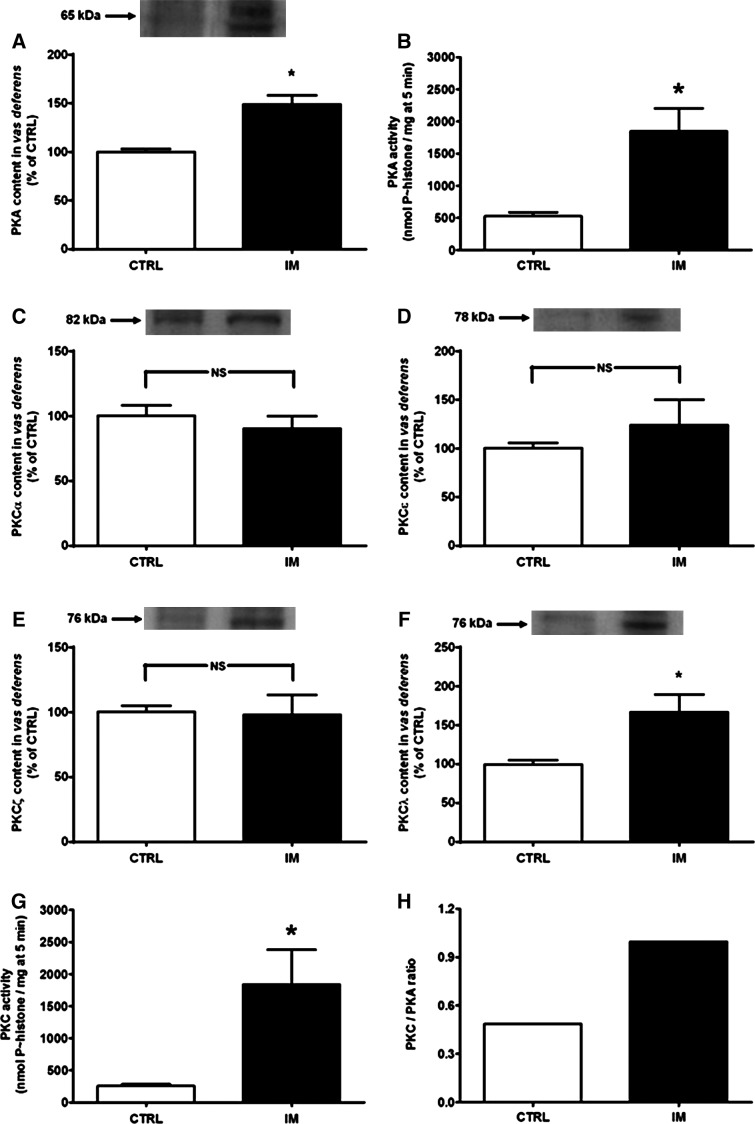
Intrauterine malnutrition upregulates PKA and PKC*λ* and decreases the PKA/PKC activities ratio. (A) PKA *α*-subunit content. Upper panel: representative immunoblotting; lower panel: graphic representation of three immunoanalyses (10% acrylamide) performed using homogenates obtained with 2–3 pairs of vas deferens from rats from different litters and a primary antibody from Santa Cruz Biotechnology (catalog number sc-903). The data are the percentages of the CTRL. *P =* *0.012 versus the corresponding CTRL. (B) PKA activity. The results are the mean ± SEM (*n *=* *4 homogenates obtained with 2–3 pairs of vas deferens from rats of different litters in both groups). *P =* *0.013 versus the corresponding CTRL. (C–F) PKC*α*, PKC*ε*, PKC*ζ*, and PKC*λ* contents. In all panels, representative immunoblotting is in the upper section; the graphic representation of four immunoanalyses performed using different homogenates obtained as above is in the bottom section. The primary antibodies were from Santa Cruz Biotechnology (catalog numbers sc-208, sc-214, sc-216, and sc-1091, respectively). *P =* *0.005 versus the corresponding CTRL. (G) Calphostin-sensitive PKC activity. The results are the mean ± SEM (*n *=* *4 different homogenates obtained as above in both groups). *P =* *0.018 versus the respective CTRL. In (A–G), the differences were assessed by unpaired Student’s *t*-test. (H) PKA/PKC activities ratio. PKA, AMP-dependent protein kinase; PKC, 4 protein kinase C; CTRL, control.

Protein kinases regulate the phosphorylation of Ca^2+^-ATPases (Baggaley et al. 2007; Masterson et al. 2011). Because undernutrition and tissue oxidative stress are associated with imbalanced PKC and PKA activity (Vieira-Filho et al. 2011, 2014), the activities and content of PKC and PKA in the vas deferens were investigated (Fig.5). The IM group had significantly increased PKA content (∼50%) and activity (∼250%) compared to the CTRL group (Fig.5A and B). Among four representative isoforms (*α*, *ε*, *λ*, *ζ*) of the three different classes of PKC (classic, novel, and atypical), only PKC*λ* was affected (∼70%); the abundance of PKCs *α*, *ε*, and *ζ* remained unmodified (compare Fig.5C–F). The upregulation of PKC *λ* was accompanied by a larger increase in total calphostin C-sensitive activity (Fig.5G). The ratio of PKC to PKA activity was two times higher in the IM group than in the CTRL group (Fig.5H).

### Intrauterine malnutrition increases the thickness of the vas deferens muscular coat in young adult rats

**Figure 6:**
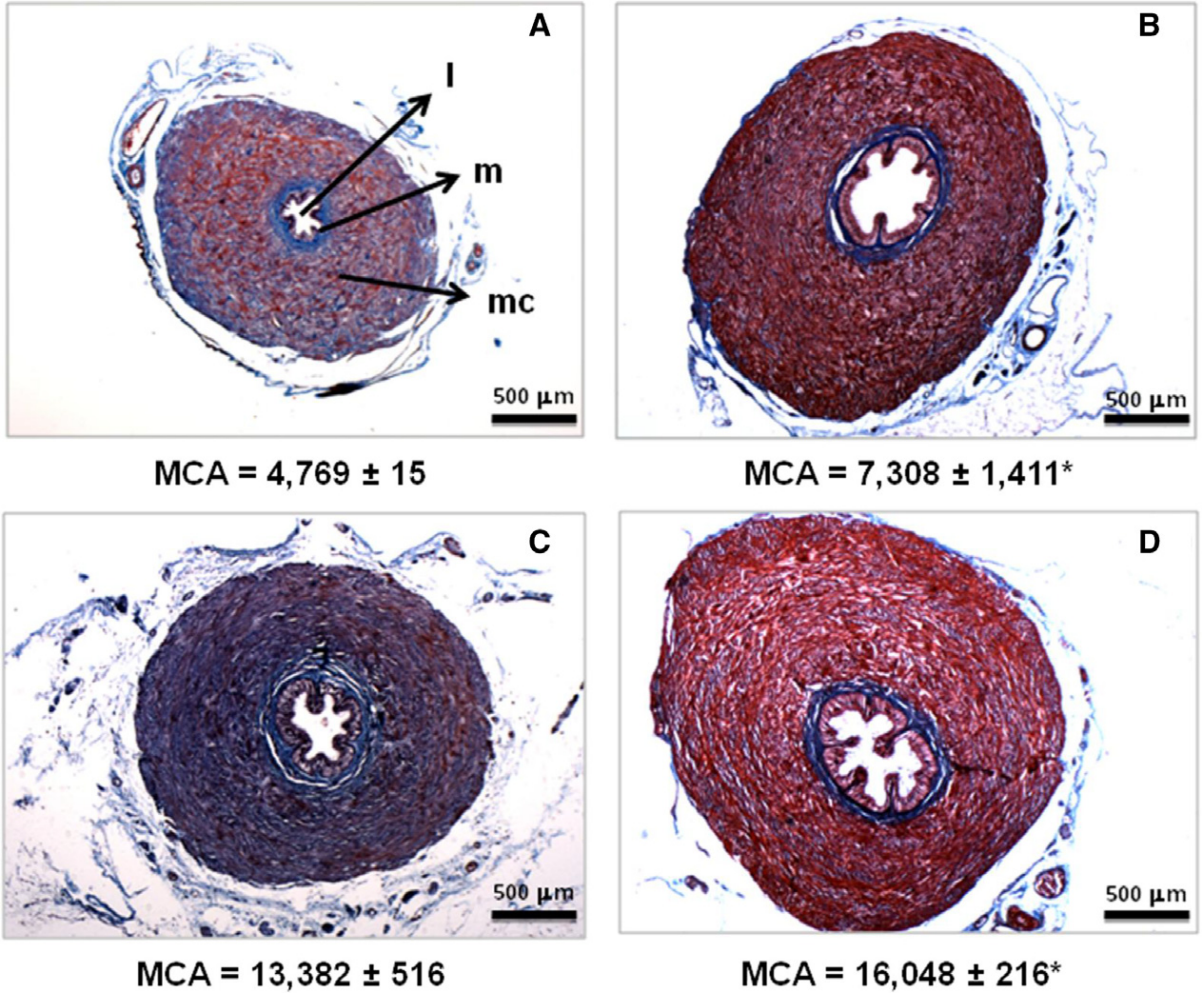
Representative photomicrographs (Masson’s trichrome) of the vas deferens. Epididymal (A and B) and prostatic (C and D) segments from CTRL (A and C) and IM rats (B and D). Key: l, lumen; m, mucosa; mc, muscular coat (keys are indicated only in A for simplicity). Representative images obtained from six different rats (all coming from different litters) from each group. The numbers below the panels (mean ± SEM in *μ*m^2^) are the corresponding values for MCA obtained from histomorphometric analyses of the six vas deferens mentioned above. *P =* *0.041 when the epididymal portion from IM rats (B) is compared with the corresponding CTRL (A); *P =* *0.0004 when the prostatic portion from IM rats (D) is compared with the corresponding CTRL (C) (unpaired Student’s *t*-test). CTRL, control; IM, intrauterine malnourished; MCA, muscular coat area.

The molecular alterations described above correlate with compromised vas deferens architecture. Masson’s trichrome-stained transverse sections taken from the initial (epididymal) and last (prostatic) quarters of the vas deferens are shown in Figure6. Histomorphometric analyses showed that the muscular coat area of the IM group was thicker in both segments (compare Fig.6A with B; Fig.6C with D). No changes were detected in the villi, the high-columnar cell shape of the epithelium, or the *lamina propria*.

### Counting of total and haploid cells in the testis, epididymis, and vas deferens

**Figure 7:**
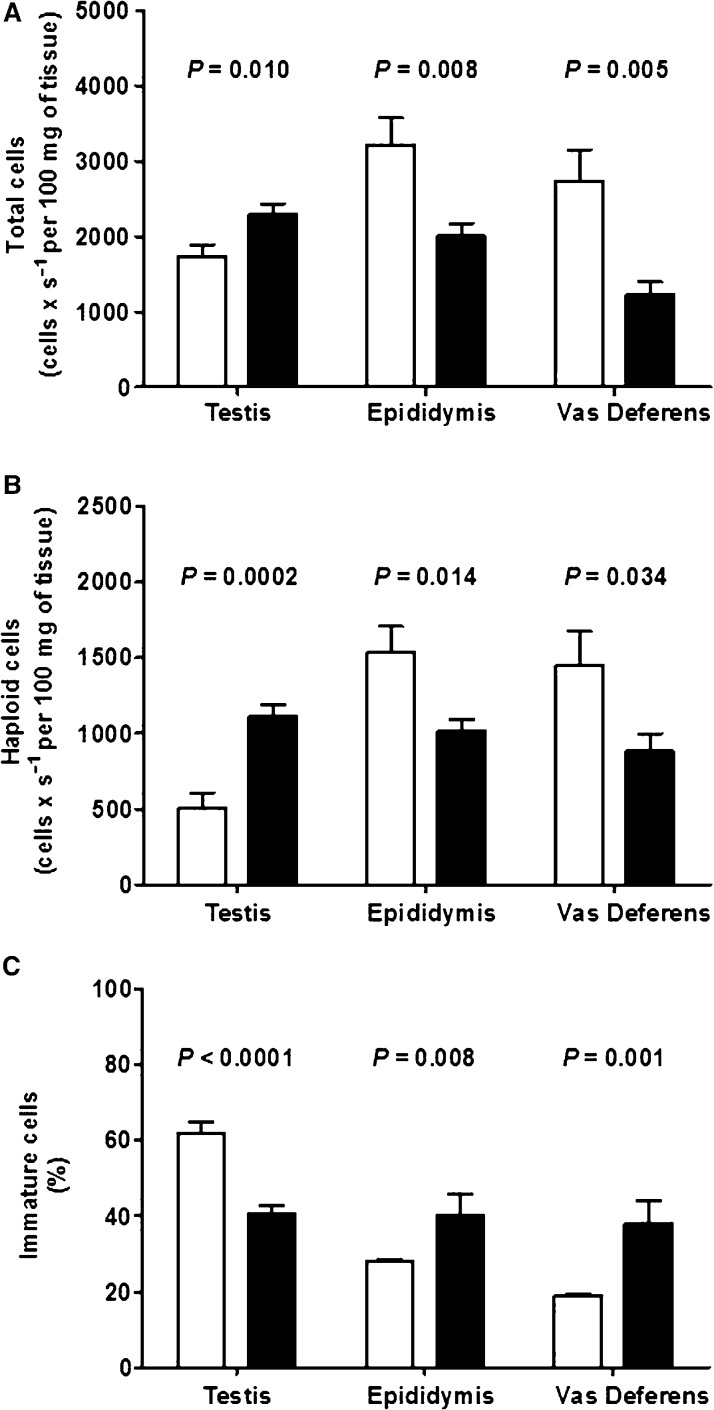
Intrauterine malnutrition affects total, haploid, and immature cell counts in the testis, epididymis, and vas deferens. Total (A) and haploid (B) cells were counted in the testis, epididymis, and vas deferens from control (empty bars, *n *=* *10 rats, all from different litters) and intrauterine malnourished rats (black bars, *n *=* *7 rats, all from different litters). (C) Percentage of immature cells in the total cell populations. The results are the mean ± SEM. *P values are indicated above the bars (unpaired Student’s *t*-test).

The following experiments assessed whether the impairment of Ca^2+^ handling and the thicker muscular coat of the vas deferens were associated with changes in the numbers of intraluminal cells. The total numbers of cells and haploid cells were monitored in the testis, epididymis, and vas deferens by measuring DNA content with flow cytometry (Fig.7). Intrauterine malnutrition increased these numbers in the testis by 35% and 130%. In contrast, these cells were reduced in number in both the whole epididymis (∼40% for total and ∼30% for haploid cells) and the vas deferens (∼55% for total and ∼40% for haploid cells) (Fig.7A and B). As expected, the opposite was observed in the number of immature cells (total minus haploid cells); there was a reduction in the testis (∼35%), an increase in the epididymis (∼40%), and a much larger increase in the vas deferens (∼130%) (Fig.7C).

### Contractile alterations of the vas deferens from adult IM rats: depressed response to phenylephrine

**Figure 8:**
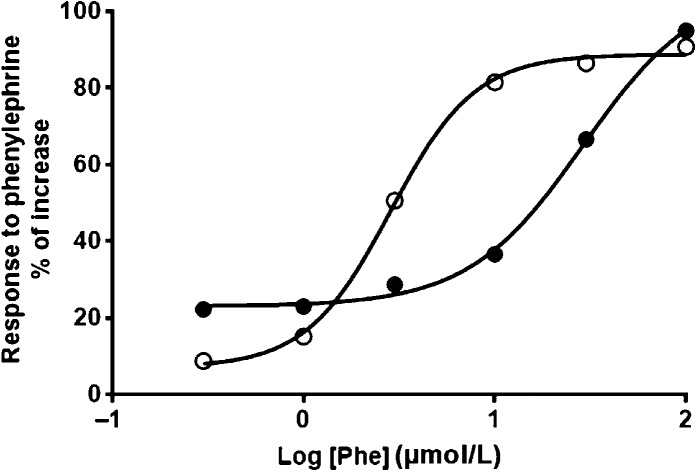
Contractile response of isolated epididymal portions of vas deferens at increasing concentrations of phenylephrine. The percent increase in contraction from baseline was recorded based on the concentrations of the *α*1-adrenoceptor agonist phenylephrine shown on the abscissa. Equation 1 (see Methods section) was adjusted to the experimental points from CTRL (○) or IM rats (●). The figure depicts the fitting of the function of the mean values. The EC_50_ and Hill slope values were calculated by averaging values obtained in each single fitting. Three to four CTRL and IM male offspring randomly selected from the litters of two CTRL and two IM dams were used, thus giving *n *=* *2 for each group. Overall, the mean values of the determinations in quadruplicate (one CTRL and one IM mother) and triplicate (one CTRL and one IM mother) agreed within 6% (CTRL pair) and 12% (IM pair). CTRL, control; IM, intrauterine malnourished.

Because the contraction of the vas deferens involves the participation of an *α*_1_-adrenoceptor-mediated signaling pathway, in which intracellular Ca^2+^ mobilization plays a central role, we investigated the response of the isolated organ to increasing concentrations of the agonist phenylephrine (Fig.8). The dose-dependence curve of IM rats was profoundly altered: the EC_50_, which averaged 2.9 *μ*mol/L in normo-nourished animals, was augmented to 28.8 *μ*mol/L. The steepness of the curves in this increasing interval, quantified by the Hill number (see eq. 1), decreased by 30% in IM rats (1.4 vs. 2.1 in CTRL).

### In utero malnutrition compromises the reproductive performance of adult male rats

**Figure 9:**
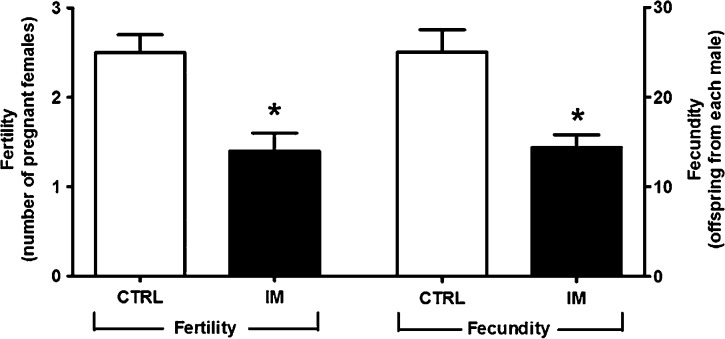
In utero malnutrition reduces the reproductive performance of adult male rats. Fertility, left axis: number of well-nourished pregnant females resulting from mating with one CTRL or one IM male rat. Fecundity, right axis: offspring from each CTRL or IM male rat. The data are the mean ± SEM of matings from six groups of one male (CTRL or IM) with three females each (fertility) or the mean ± SEM of offspring from six mated groups with one CTRL or one IM male rat (fecundity); *n *=* *6, corresponding to males (CTRL or IM) coming from different litters, originally with eight pups each. *P =* *0.002 (fertility) and *P =* *0.005 (fecundity) versus the corresponding CTRL (unpaired Student’s *t*-test). CTRL, control; IM, intrauterine malnourished.

Figure9 shows that fewer normo-nourished females became pregnant after mating with IM males (1.4 ± 0.2) compared with CTRL males (2.5 ± 0.2). As a consequence, the total offspring from each male fell from 25.0 ± 2.5 (CTRL) to 14.4 ± 1.4 (IM). The males could have been subfertile because fewer sperm cells reached the upper female tract and oocytes. However, this does not seem to be the case because there was no difference in the number of pups per dam, regardless of whether the mothers had become pregnant after mating with CTRL or IM males (average 10 pups per litter). Thus, the impairment in reproductive performance is exclusively related to the male offspring that had suffered from intrauterine malnutrition.

## Discussion

We clearly demonstrate that the vas deferens responds differently to malnutrition, depending on whether it occurs during the narrow window of gestation or after chronic undernutrition throughout life (Muzi-Filho et al. 2013). There are specific mechanistic events and structural/functional consequences that reinforce the importance of this life stage in the undernutrition-induced lesions encountered in the same organs and tissues (Fowden et al. 2006). The most important differences between placental and chronic undernutrition (with the same diet) in terms of impact on vas deferens are related to the following: (1) passive permeability of the epithelium to Ca^2+^; (2) mechanisms and targets that increase oxidative stress; (3) reversed imbalanced PKC/PKA activity ratios; (4) the possibly unique role of programmed PKC*λ* upregulation in male rats undernourished in utero; and (5) differential impact on the organ architecture, as discussed below.

Intrauterine undernutrition in pregnant females culminated in the impairment of the reproductive capacity of young adult male offspring, as assessed by reproductive performance. Although this assessment was quite simple, a 50% decrease in the number of pregnant females and offspring from each male gives a general idea about the compromised reproductive status. Some evidence helps to substantiate the dominance of both the poor quality and quantity of ejaculate as the result of fetal undernutrition. Similar to that experienced in this study, gestational protein restriction to a low level previously resulted in reduced Sertoli cell and sperm numbers and the impairment of sperm motility and morphology in adult males (Toledo et al. 2011).

The imprinting of molecular changes at the level of intracellular Ca^2+^ homeostasis in the vas deferens*,* which occurred in a very narrow window of 3 weeks of gestation, programmed a legacy that affects one of the most important physiological functions of living beings in adulthood age – the preservation of reproductive potential and species perpetuation. These findings increase our understanding of the developmental programming of physiological systems, which mostly focuses on cardiovascular disorders and predisposition to metabolic diseases (Gluckman and Hanson 2004; Brenseke et al. 2013). Although pioneering studies on protein restriction during gestation have shown hormonal alterations that impair reproductive capacity (Faria et al. 2008; Kotsampasi et al. 2009; Genovese et al. 2010), the molecular mechanisms influencing an altered phenotype of offspring at a key reproductive target, such as the vas deferens, remain largely undetermined.

The vas deferens is of particular interest because the smooth muscle of the vas deferens walls reflexively contracts during the ejaculatory process, thus propelling sperm from the epididymis to the prostatic urethra (Westfall and Westfall 2001; Koslov and Andersson 2013). This contraction is dependent on a motile Ca^2+^-dependent process (Scaramello et al. 2009) observed in other smooth muscles (Floyd and Wray 2007). The molecular entities responsible for Ca^2+^ homeostasis in the vas deferens were reported over a decade ago (Scaramello et al. 2002). Ca^2+^ leakiness, observed as a reduction in accumulation (Fig.1), may be associated with an undernutrition-induced decrease in FKBP12 (Fig.3C) together with clearly uncoupled, and therefore inefficient, Ca^2+^-ATPase activity (Fig.2); these data support the hypothesis that malnutrition provokes an improper response in the adult to adaptive mechanisms triggered at an early age. The upregulation of SERCA2 and its accelerated catalysis might be an attempt to provide a sufficient supply of Ca^2+^ to the contractile machinery of vas deferens cells. The significantly thickened muscle wall in the two portions of the vas deferens (Fig.6) matched its increased mass (Table3), which can be viewed as a compensatory early adaptive mechanism, but it did not sufficiently prevent the strongly altered physiological behavior of the muscle machinery in adult life, as confirmed by its depressed contractile response to phenylephrine (Fig.8). The imprinted changes in Ca^2+^ transporters could have thus led to totally disrupted Ca^2+^ handling in adult life and the impairment of muscle contraction.

The disruption of bodily Ca^2+^ homeostasis is one important consequence of protein restriction during pregnancy due to impaired renal Ca^2+^ handling (Ashton et al. 2007), an effect that persists throughout the offspring’s life. Possibly, and despite the preserved plasma ionized Ca^2+^, the vas deferens was adversely impacted in terms of Ca^2+^-modulated processes, as demonstrated for bone (Ashton et al. 2007). Alterations in ionic transport processes – possibly those of Ca^2+^ – could also have affected the normal regulation of the luminal environment to which sperm cells are exposed (Pierucci-Alves et al. 2009, 2010). This proposal is supported by the observation that intracellular Ca^2+^ seems to be a key regulator of transepithelial fluxes in the efferent ducts and epididymis (Belleannée et al. 2009), as it is in other epithelia (Jung and Lee 2014). It is noteworthy that the RBD culminated in strongly reduced steady-state Ca^2+^ transport in vesicles derived from vas deferens homogenates only when administered to pregnant rats (Fig.1), but not when given directly throughout life after weaning (Muzi-Filho et al. 2013). This difference indicates increased Ca^2+^ permeability and possibly the different exposure of contractile proteins to the cation in programmed males. Moreover, maternal undernutrition could have imprinted alterations in water and solute fluxes, including the flux of H^+^ (thereby changing pH) and glycerol – a substrate for sperm cells – as the result of a direct influence on water channels and H^+^ pumping mechanisms mediated by a V-type ATPase and regulated by Ca^2+^ (Belleannée et al. 2009). Measurements of intraluminal pH along the different segments of the vas deferens would be helpful for localizing specific lesion points.

The preservation of serum and intratesticular testosterone, as observed with a protein-deficient diet (Toledo et al. 2011), could help preserve sperm production. Abnormal downstream alterations in the vas deferens that alter the mechanism of cellular transport could also contribute to the increase in cell numbers in the testis, providing evidence that opposite (both beneficial and deleterious) processes can be imprinted by intrauterine malnutrition. In the face of recent observations demonstrating that luminal content composition indirectly affects the success of embryonic survival (Bromfield et al. 2014), we surmise that maternal undernutrition could also cause imprinted alterations in signaling pathways related to the preservation of male gametes for normal fecundity. With this view, it is possible that decreased fertility and fecundity (Fig.8) could be related to other factors, such as signaling agents (Bromfield et al. 2014), in addition to those that disturb Ca^2+^ handling, muscle contractility, and the propulsion of cells along the lumen of the vas deferens. The participation of other factors, such as decreased sperm quality (higher number of immature cells, for example; see Fig.7C), structural changes in the testis and epididymis, and alterations in the composition of luminal fluid, could have also contributed to decreased fertility. These alterations, programmed by maternal undernutrition and acting alone or in association, could be affected by the timing of sperm production and transport from the testis and epididymis to the end of the vas deferens. It may be that inflammation, hypoxia, and oxidative stress (see below) can mimic and exert comparable effects to the broad ensemble of toxicants involved in the pathogenesis of male reproductive disturbances (for an extensive review, see Creasy 2001). Therefore, it is clear that the testicular and epididymal effects of maternal undernutrition in male adult offspring should be more carefully and directly investigated.

Increased oxidative stress is a hallmark of undernutrition status under different conditions (Vieira-Filho et al. 2009, 2011; Muzi-Filho et al. 2013). For this reason, we chose to investigate two key alterations in the context of ion transport (including Ca^2+^): membrane lipid peroxidation and the carbonylation that especially impacts Ca^2+^ transporters (Strosova et al. 2011). Perinatal undernutrition due to RBD, with early elevated oxygen reactive species (ROS) that progressively decrease, results in an increased abundance of renal regulatory subunits of NADPH oxidase during adulthood (Vieira-Filho et al. 2014) as an imprinted marker of this previous high ROS production. The increased generation of ROS leads to sperm DNA damage and reduced reproductive capacity (Aitken and Krausz 2001). The evolving scenario regarding ROS seems to be similar in the vas deferens, where lipid peroxidation levels are similar in CTRL and IM rats (Fig.4A), suggesting that this phenotype is reversible as a consequence of consuming a normal diet after weaning. Conversely, chronically undernourished males present with very high levels of lipoxidation products, possibly as a result of continuous undernutrition inducing oxidative stress.

In the in utero malnutrition model, increased carbonylation-induced protein oxidative damage without lipid peroxidation or alterations of -SH groups (Fig.4B and C) is evidence of the perpetuation of a specific oxidative pathway known to affect Ca^2+^ transporters (Strosova et al. 2011). The idea of a relatively specific mechanism of RBD-induced oxidative damage in the vas deferens is supported by the observation that this same profile of oxidative stress occurs in male rats chronically undernourished in this dietary model (Muzi-Filho et al. 2013). Similar levels of protein carbonylation coexisting with different lipid peroxidation levels are indicative of an important mechanistic difference that depends on the diet administered at different stages of development. Protein carbonylation without lipid peroxidation in vas deferens membranes of male rats undernourished in utero indicates direct oxidation of amino acid side chains (Møller et al. 2011). As an important difference, an indirect mechanism mediated by lipid peroxidation products (Fritz and Petersen 2011) appears to participate with chronic undernutrition. These two distinct routes can determine the direction, type, and velocity of pathological processes (Dalle-Donne et al. 2006). The IM diet could also have programmed altered adiposity, which could increase low-grade inflammation and additional oxidative stress in the male reproductive tract. Low-grade inflammation caused by adipocyte dysfunction (altered secretion of cytokines) could lead to hypoperfusion, local hypoxia, and increased ROS production (Netzer et al. 2015), a widespread association that, interestingly, is also able to impact female reproductive organs (Spritzer et al. 2015).

An unbalanced regulatory interaction between PKC and PKA (with a relative increase of the former) is a major consequence of uncontrolled tissue stress at the molecular signaling level due to undernutrition (Luzardo et al. 2011; Vieira-Filho et al. 2011, 2014; Silva et al. 2014a). Interestingly, an increased calphostin-sensitive PKC/PKA activity ratio seems to be underpinned by an accelerated turnover of PKC isoforms sensitive to calphostin rather than by their contents. An increase of the atypical PKC*λ* isoform, which is insensitive to calphostin (Steinberg 2008), might also be a vestige of this same evolving oxidative process. Although its role in male reproductive biology is unknown, its presence as a marker of undernutrition-linked oxidative stress is clear in the adult kidney (Vieira-Filho et al. 2011). PKC*λ* is involved in modulating oxidative damage in other epithelia (Farhadi et al. 2006); from its unique upregulation in the vas deferens, it is speculated that it also participates in the functional impairment of this organ. The decreased PKC/PKA ratio found in chronic undernutrition (Muzi-Filho et al. 2013) – another important mechanistic difference – indicates that the impact on kinases modulating Ca^2+^ transporters (Assunção-Miranda et al. 2005; Di Leva et al. 2008; Axelband et al. 2012) is critically dependent on the window during which dietary restriction is imposed. This time dependence suggests that the phosphorylation mechanisms ultimately leading to disturbed Ca^2+^ transport in the vas deferens differ in young adult rats undernourished at the beginning or throughout life. The significance of these differences in terms of reproductive performance remains to be elucidated.

In conclusion, this study presents experimental evidence regarding the molecular mechanisms involved in disturbed vas deferens function in adulthood (Fig.8), which could contribute to compromised adult male reproductive capacity, and thus fertility and fecundity, in rats exposed to intrauterine malnutrition (Fig.9). Findings at the cellular, molecular, and regulatory levels give support to the view that in human populations, dietary restriction in early life might not allow offspring to develop their reproductive potential in harsher environments in later life (Hayward et al. 2013). Cellular disturbances (including Ca^2+^ handling in the vas deferens) associated with early nutritional restriction persist in adult life, even when receiving a normal diet; the male genital tract is permanently deficient in function, as revealed by the compromised contractile activity of the vas deferens.
